# Degradation of chlorpyriphos and polyethylene by endosymbiotic bacteria from citrus mealybug

**DOI:** 10.1016/j.sjbs.2021.03.058

**Published:** 2021-03-27

**Authors:** Shahida Ibrahim, Rakesh Kumar Gupta, Abdul Rasheed War, Barkat Hussain, Amit Kumar, Tariq Sofi, Ahmad Noureldeen, Hadeer Darwish

**Affiliations:** a Division of Entomology, SKUAST-J, Chatta-180009, Jammu, Jammu and Kashmir, India; b World Vegetable Center, ICRISAT Campus, Patancheru-502324, Hyderabad, Telangana, India; c Division of Entomology, SKUAST-K, Shalimar Campus-190025, Srinagar, Jammu and Kashmir, India; d Instrumentation Division, Indian Institute of Integrative Medicine, Canal Road-180001, Jammu, Jammu and Kashmir, India; e Division of Plant Pathology, SKUAST-K, Shalimar-190025, Srinagar, Jammu and Kashmir, India; f Department of Biology, College of Sciences, Taif University, P.O. Box 21944, Saudi Arabia; g Department of Biotechnology, College of Sciences, Taif University, Taif 21944, Saudi Arabia

**Keywords:** Mealybug bacterial endosymbionts, Chlorpyriphos, Polyethene degradation

## Abstract

Chlorpyriphos is one of the major organophosphorus pesticides used widely to control a range of insect pests across several crops. This insecticide is hazardous to the environment and toxic to mammals, thus, it is essential to remove the same from the environment. Similarly, use of polythene is also increasing day by day. Therefore, it is highly important to identify ways to degrade chlorpyriphos and other pesticides from the environment. We studied the degradation of chlorpyriphos and polyethylene by Citrus mealybug (*Planococcus citri*) bacterial endosymbionts such as *Bacillus licheniformis, Pseudomonas cereus, Pseudomonas putida* and *Bacillus subtilis*. This investigation revealed that bacterial endosymbionts use the polythene as a source of carbon and solubilize them by their enzymatic machinery. The degradation of polyethylene by endosymbionts showed a significant reduction in weight of polyethylene sheet after 15, 30 and 45 days of treatment. The SEM images showed localized degradation of the polyethylene around the bacterial cells in the biofilm. Further, the tensile strength (percentage elongation) was significantly reduced after 45 days of incubation. The weight of paraffin wax showed significant reduction in *B. cereus*. A significant reduction in total amount of chlorpyriphos in soil was observed at an interval of 7, 14 and 21 days after treatment by the bacterial isolates. Among the bacteria, *B. cereus* and *P. putida* were found to be most effective. The results from this study show that endosymbionts can be significantly implicated in degrading chlorpyriphos and polyethylene from the environment.

## 1 Introduction

Mealybugs (Hemiptera: Pseudococcidae) are small, soft-bodied insects containing more than 2000 described species in 290 genera, globally (Downie and Gullan, 2004). They feed on more than 200 plant species of fruits, vegetables and grasses, including grape, fig, date palm, apple, avocado, banana, citrus, okra, tomato, brinjal, cotton, sugarcane, pulses and a few ornamentals (Miller et al. 2002). They suck plant sap causing wilting, distorting and yellowing (chlorotic) of leaves. Under severe infestation, the attacked plants show premature leaf drop, stunted growth and may lead to death of infested plants or plant parts (Kosztarab and Kozar, 1988, Nagrare et al., 2009). Further, mealybugs excrete honeydew on which sooty mould develops, which causes darkening of the attached parts, leading to reduced fruit quality and photosynthetic rate (Godfrey et al., 2002). The reproductive rate of mealybugs is high, and they hide in the cracks, cervices and corner regions of plants. They spread through wind, water, rain, birds, human beings and farm animals. Mealybugs have developed resistance to several insecticides, which is mostly associated with the endosymbionts (bacteria, fungi) in the insect gut (Munson et al., 1992, Kantheti et al., 1996). The endosymbionts produce enzymes that detoxify insecticides (Kantheti et al., 1996). Many researchers have isolated mealybug endosymbionts for bioremediation of insecticide, polyethylene and waxes (Gatehouse et al., 2012, Kantheti et al., 1996, Munson et al., 1992).

The organophosphorus group of pesticides (OP) is the highly toxic group of pesticides, and accounts for about 38% of the total pesticides globally (Singh and Walker, 2006). Chlorpyriphos is a broad-spectrum insecticide used against several insect pests on a range of crops (Maya et al., 2011). Its slow degradation leads to the contamination of water and soil and cause other health issues as well (Chishti et al., 2013). The biodegradation i.e., the degradation of materials by microbes such as bacteria, fungi and algae is an environmentally friendly degradation of pesticides (Abatenh et al., 2017).

Bioremediation uses living microorganisms to degrade the pesticides into less toxic forms and depends on the type of pesticide, environmental matrix and the organisms present in the ecosystem (Abatenh et al., 2017). Endosymbiont, *Burkholderia* found associated with stinkbugs, resulted in the biodegradation of organophosphorus pesticides (Tago et al., 2014). Bacterial isolates from mealybugs have been identified as *Bacillis licheniformis, Pseudomonas cereus, Pseudomonas putida* and *Bacillus subtilis* and were cultured for degradation of toxic chlorpyriphos and polyethylene. Insecticide degradation by these endosymbionts has been studied in detail and these isolates have been found to grow in a selective medium supplemented with substantial amount of chlorpyriphos and paraffin wax (Blanton and Peterson, 2020). We studied the role of diverse mealybug endosymbionts in degradation of chlorpyriphos and polyethylene.

## 2 Materials and methods

The present study was conducted in the Biocontrol Laboratory, Division of Entomology, Sher-e-Kashmir University of Agricultural Sciences & Technology, Main Campus, Chatha, Jammu.

### 2.1 Sample collection

*Planococcus citri* colonies of adult females collected from the Sher-e-Kashmir University of Agricultural Sciences and Technology, Main Campus, Chatha, Jammu, (32° 44′ N/74° 54′ E), Jammu and Kashmir, India. The samples were collected from the infested okra plants and transported to the biological control laboratory in sealed polythene bags.

### 2.2 Mealybug rearing

A disease-free laboratory colony of mealybugs, *Planococcus citri* was reared and maintained on Indian baby pumpkins, *Praecitrullus fistulosus* (stocks) (Cucurbitales: Cucurbitaceae). The colony was maintained by releasing one female per pumpkin, kept in rearing cages made of wood measuring (45 × 45 × 45 cm, LxBxH) at 37 ± 2 °C, 70 ± 5% RH, and photoperiod (16:8h) light: dark.

### 2.3 Chemicals

A laboratory grade chemicals, media and solvents were purchased from HiMedia, Mumbai, India. Glassware, media and other materials were autoclaved at 121 °C at 15psi/sq. inch for 15 min according to laboratory protocol and the preparation of reagents as per the protocol instructions.

### 2.4 Endosymbiont bacteria culture

*Planococcus citri* adults obtained from the laboratory culture were anaesthetized using CO_2_ and subsequently killed on dry ice for one minute and frozen in refrigerator. Isolation and purification of potential endosymbionts associated with *P. citri* was carried out from the cadavers of adult mealy bug as per Ibrahim et al. (2020). The isolated colonies developed in some regions of the plate were picked using sterile loop and repeatedly sub-cultured by streak inoculation until single cell colonies were obtained. Pure cultures of the endosymbiont bacteria associated with mealy bug were maintained on nutrient agar slants at 4 °C for further analysis.

### 2.5 Preparation of polyethylene and insecticide (chlorpyriphos) degrading bacterial inoculums

The pure culture of selected bacterial isolates namely *Bacillus subtilis, Pseudomonas putida*, *Bacillus licheniformis* and *Bacillus cereus* were cultured from the Entomology and bacteriology Laboratories of SKUAST-Jammu, Jammu and Kashmir, India and inoculated separately in 50 ml nutrient broth in a 250 ml capacity flasks and incubated at 37 °C for 24 h in a rotary shaker incubator at 200 rpm. The 24 h old cultures were centrifuged at 4,225 rpm for 10 min and the cell pellets were diluted with 10 ml sterile saline solutions separately. The viable cell count was determined by the plate count method (Perscott et al., 2005). The cell numbers of each isolate were standardized to approximately at 1 × 10^8^ cells ml^−1^.

### 2.6 In vitro degradation potential of selective strain for chlorpyriphos and polyethylene

#### 2.6.1 Dissipation of chlorpyriphos in liquid media (Nutrient broth) by different bacteria

To study the degradation of chlorpyriphos, a stock culture of each endosymbiont (bacteria) was grown in nutrient medium for 48 h to mid-log phase of growth. A 70 μl of chlorpyriphos (1%) prepared in acetone was added in a pre-sterilized 100 ml Erlenmeyer flask containing 50 ml of nutrient broth and shaken for 2 h. The flask was added with 48 h old cell suspension of endosymbionts grown on nutrient media, centrifuged at 8,000 rpm for 10 min and the precipitate was resuspended in sterile distilled water to obtain a final density of about 1 × 10^8^ CFU (colony forming units) ml^−1^. Medium uninoculated with a bacterial suspension served as a control. Both inoculated and uninoculated samples were incubated under intermittent shaking to provide aerobic conditions. After 7, 14, and 21 days, duplicate flasks from inoculated and uninoculated samples was withdrawn aseptically, cleaned and analyzed for pesticide residues by High Performance Liquid Chromatography at the IIIM, Jammu, India.

Degradation percentage was determined by using following equation:

$$\mathrm{Degradation}\;(\%)=\frac{\mathrm{Residual}\;\mathrm{amount}\;\mathrm{in}\;\mathrm{blank}-Residual\;amount\;in\;sample}{\mathrm{Residual}\;\mathrm{amount}\;\mathrm{in}\;\mathrm{blank}}\times 100$$

#### 2.6.2 Clean up procedure

A 10 g of homogenized/hydrated sample was taken in a 50 ml centrifuge tube, to which 10 ml of Acetonitrile was added. To this, 4 g MgSO_4_ and 1 g NaCl were added and the mixture was shaken vigorously for 1 min, then centrifuged at 5000 rpm for 5 min. The aliquot (1 ml) of the supernatant was transferred to a micro centrifuge tube containing 150 mg MgSO_4_ and 50 mg primary secondary amine (PSA). After shaking for 1 min, the reaction mixture was centrifuged at 6000 rpm for 1 min. The supernatant (0.5 ml) was used for HPLC analysis. Rest of the sample was stored at 2 ℃ for further analysis.

#### 2.6.3 Effect on residual weight of polyethylene

Films of low density polyethylene (LDPE) were washed with alcohol, rinsed with sterilized distilled water and dried. Pre sterilized disks of LDPE sample were aseptically transferred into a conical flask containing 50 ml of nutrient broth (pH 7). Inoculum of screened polyethylene degrading bacteria was inoculated into the same flask containing 50 ml of nutrient broth with pre-weighed disks of low-density polyethylene, maintained at 30 ℃ in a rotary incubatory shaker for 45 days. Two sets of experiments were maintained i.e., control (media + low density polyethylene) and treated (media + low density polyethylene + bacterial inoculum). All experiments were run simultaneously in triplicate. Bacterial growth was checked by a UV-Vis spectrophotometer (Shimadzu UFLC, Kyoto, Japan) at 600 nm.

#### 2.6.4 Dry weight determination

The treated and untreated polyethylene discs were recovered after 45 days of incubation. After washing with aqueous sodium dodecyl sulphate (2%) for 4 hrs followed with distilled water, the discs were washed with 70% ethanol to ensure maximum possible removal of cells and debris. The washed LDPE film was placed on a filter paper and dried overnight at room temperature before weighing.

Degradation percentage was determined by using following equation:

$$Degredation\;\left(\%\right)=\frac{Initial\;weight-Final\;weight}{Initial\;weight}X100$$

#### 2.6.5 Scanning Electron Microscopy

The untreated and treated samples after 45 days of duration were subjected to SEM imaging (after washing with aqueous Sodium Dodecyl Sulphate (2%) and distilled water, repeatedly and additionally flushed with ethanol (70%) with the objective of removal of cells so as to get maximum surface to be exposed for visualization. The samples were pasted onto the SEM Sample Stub using a carbon tape and sample was coated with gold, palladium for 40 s and analyzed under high-resolution SEM (JEOL, Model JSM-6390LV). SEM analysis was carried out to investigate micro cracks, pits, erosion of surface, cracking and polymer adhesion.

#### 2.6.6 Measurement of tensile strength of polyethylene

The tensile strength of the samples was measured at the Government Polytechnic College, Jammu, India. Polyethylene sheets treated and untreated samples were recovered after 45 days of incubation. After washing with aqueous sodium dodecyl sulphate (2%) for 4 hrs followed with distilled water, the samples were washed with ethanol (70%) to ensure maximum possible removal of cells and debris. The washed LDPE film was placed on a filter paper and dried overnight at room temperature and the tensile strength was measured as N/mm^2^.

#### 2.6.7 Residual weight of liquid paraffin

Liquid paraffin was used as a carbon source for all bacteria. Liquid paraffin (5 mg) was poured in all test tubes containing bacterial inoculum and control tubes. After 30 days of inoculation, all the test tubes were examined for change in the residual weight (g) of liquid paraffin.

#### 2.6.8 Effect of different bacterial species on residual weight of solid wax

Equal amount of solid wax (2.54 g) was placed on cover slips and each coverslip having solid wax was placed in petriplate containing nutrient agar. Each coverslip was inoculated by bacterial isolate, control was without inoculum. After 30 days of inoculation, weight of solid wax was measured to check the changes in the residual weight of solid wax. The weight of solid wax was expressed in mg.

### 2.7 Degradation of insecticide in soil and water through bio augmentation

#### 2.7.1 Collection of soil and water samples

Insecticide free, soil and water samples were collected. The soil and water samples were tested in the laboratory for any insecticide. The said soil and water samples were assigned to the treatments as under:

1. Control (samples containing only insecticide)
2. Treated (samples containing insecticide and culture of bacteria)

#### 2.7.2 Degradation in soil

A 50 ml soil slurry was prepared in a 100 ml Erlenmeyer flask. A known quantity of inoculum (1 ml) was then added to the treatments. Subsequently, the procuring bacteria were bioaugmented in soil samples. Chlorpyriphos 1% (110 µl) prepared in acetone was added to 100-ml flasks containing soil as media and incubated at 37 °C in an incubator. The degradation of these samples was analysed by collecting these samples after 7, 14 and 21 days of incubation by the High Performance Liquid chromatography technique after cleaning of sample. The amount of chlorpyriphos was quantified by HPLC.

#### 2.7.3 Dissipation in water

Insecticide free water samples (50 ml) were collected in 100 ml flasks. The bacterial inoculum (1 ml) was added to the water samples. The procuring bacteria were bioaugmented in water samples. Chlorpyriphos 1% (160 μl) in acetone was added to the water sample. Water samples with bacterial inoculum but without insecticide were maintained as control. All the samples were incubated at 37 °C. The degradation of these samples was analysed after 7, 14 and 21 days of incubation by HPLC.

### 2.8 Statistical analysis

The data were subjected to analysis of variance (ANOVA) using SPSS v15.1 (SPSS, Inc., Chicago, IL, USA). The means were separated by Tukey’s test when the treatment effects were statistically significant (p ≤ 0.05).

## 3 Results

### 3.1 Dissipation of chlorpyriphos in liquid media by different bacteria

**Fig. 1:**
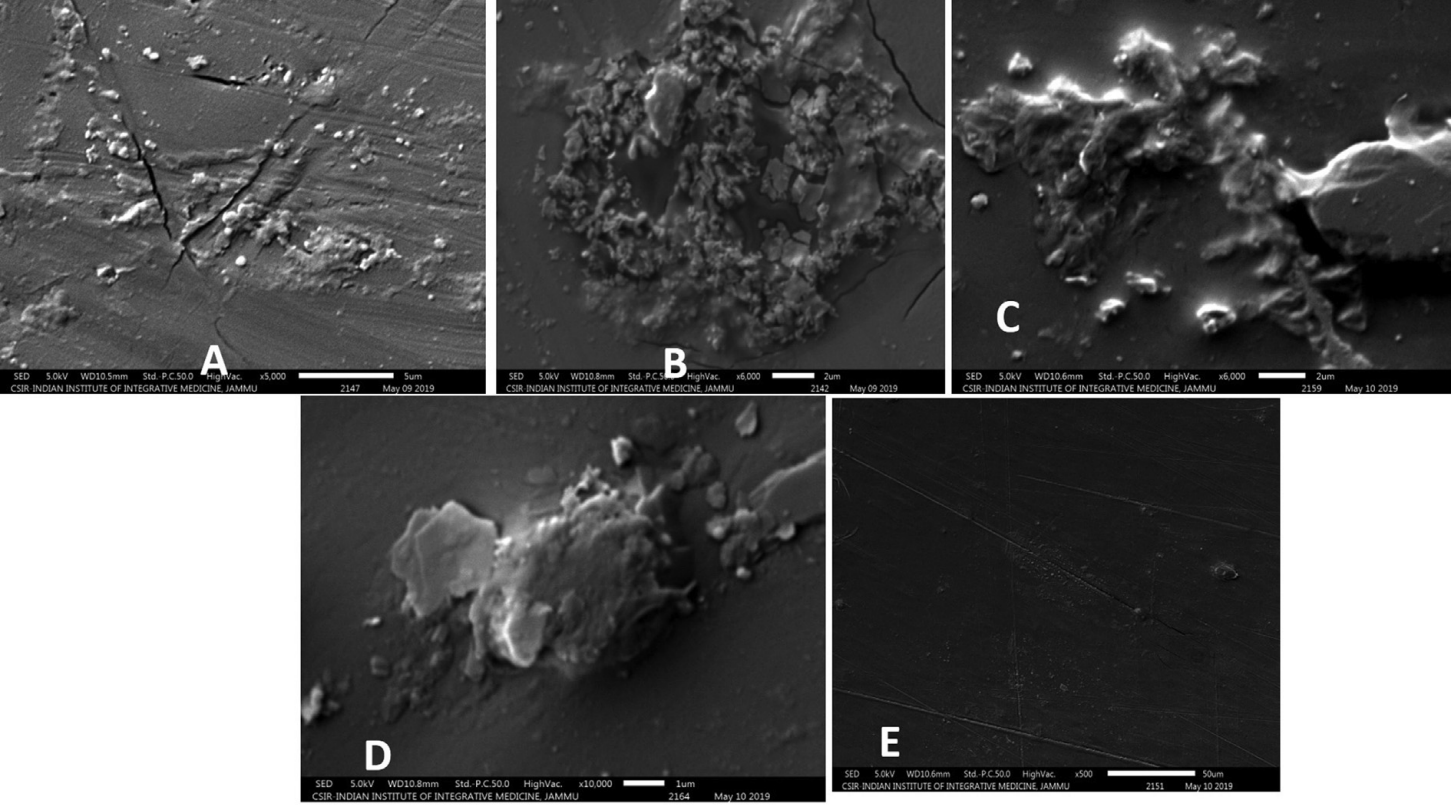
Changes in morphological characters by Scanning Electron Microscopy (SEM) A: *Pseudomonas putida* B: *Bacillus licheniformis* C*: Bacillus cereus* D*: Bacillus subtilis* E*:* Control*.* Note: We see that the control film is smooth, while after 45 days’ incubation roughness of film increases which confirms degradation of film by bacterial isolates.

The chlorpyriphos in liquid media was analysed for its dissipation. As compared to control, all the treatments showed significant reduction in the amount of chlorpyriphos in liquid media at 7 days after incubation (F = 2.955, df = 4, 10, p = 0.000), 14 (F = 2.622, df = 4, 10, p = 0.000) and 21 (F = 1.950, df = 4, 10, p = 0.000). While isolated bacteria *B. cereus* and *B. subtilis* could bring total dissipation of chlorpyriphos, *P. putida* also reduced it to 69.696% at 21 days after incubation (Table 1, Supplementary 1–5). A significant increase in dissipation rate was observed with an increase in time interval within bacterial species i.e., *B. subtilis* (F = 1.529, df = 2, 6, p = 0.000), *P. putida* (F = 2.047, df = 2, 6, p = 0.000), *B. licheniformis* (F = 3.557, df = 2, 6, p = 0.000), *B. cereus* (F = 8.081, df = 2, 6, p = 0.000) (Fig. 1).

**Table 1:** Dissipation of chlorpyriphos in liquid media by different bacteria.

**Treatments**	**Days after treatment**					
	**7**		**14**		**21**	
	**µg/20 µl**	**Loss (%)**	**µg/20 µl**	**Loss (%)**	**µg/20 µl**	**Loss (%)**
**Control**	0.275 ± 0.0006^e^	1.428	0.275 ± 0.0006^d^	1.428	0.263 ± 0.0006^d^	5.714
***Pseudomonas putida***	0.201 ± 0.0007^d^	27.173	0.161 ± 0.0010^c^	46.666	0.080 ± 0.0006^c^	69.696
***Bacillus cereus***	0.078 ± 0.001^a^	71.739	0.037 ± 0.0006^a^	86.594	0.000 ± 0.0000^a^	100.000
***Bacillus licheniformis***	0.176 ± 0.001^c^	36.231	0.161 ± 0.0010^c^	46.666	0.002 ± 0.0006^b^	99.242
***Bacillus subtilis***	0.148 ± 0.0006^b^	46.376	0.054 ± 0.0017^b^	80.434	0.000 ± 0.0000^a^	100.000

Mean ± SE in the same column followed by different letters are significantly different (*Tukey* HSD, p < 0.05), SE = standard error of means.

### 3.2 Effect on residual weight of polyethylene

**Fig. 2:**
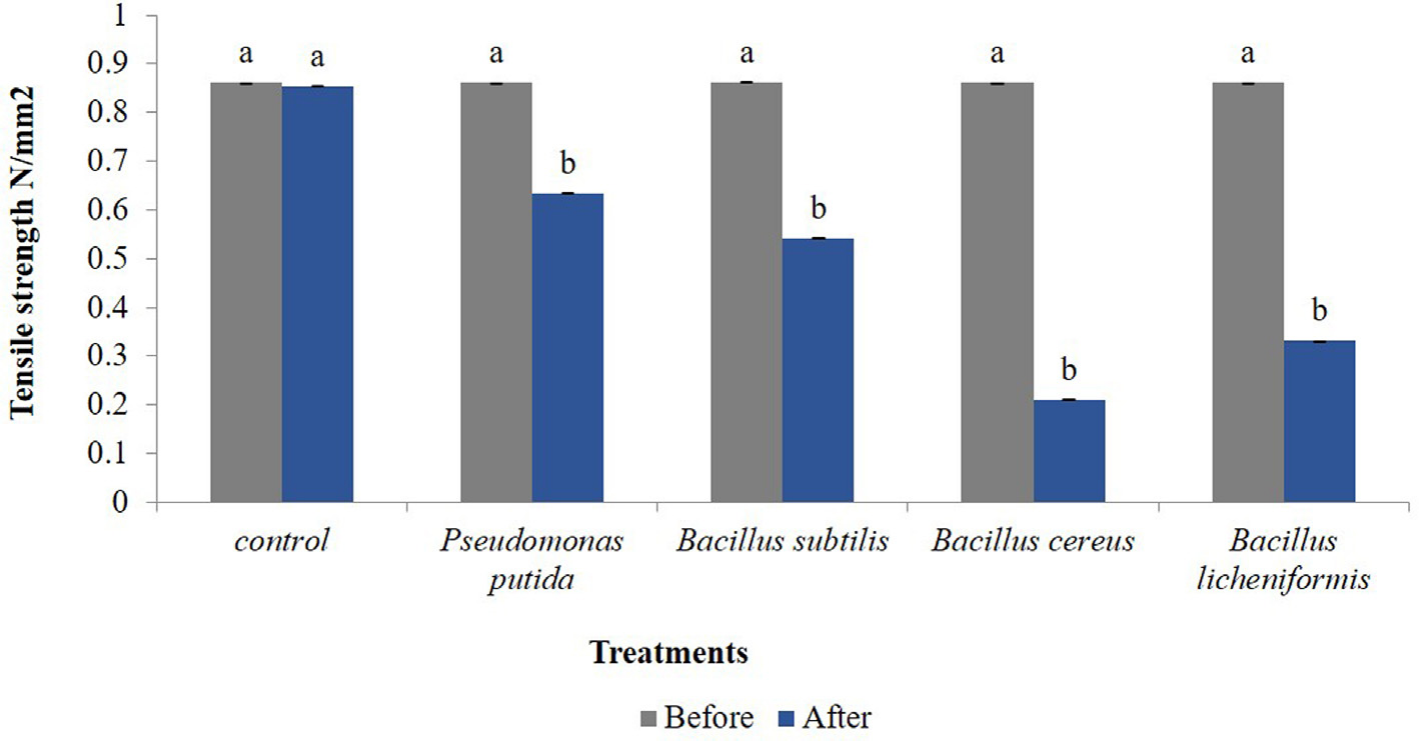
Tensile strength (N/mm^2^) of polyethylene of control and treated samples after 45 days of incubation*.* Bars with similar letters within a treatment are not statistically different (*Tukey* HSD, p < 0.05).

**Fig. 3:**
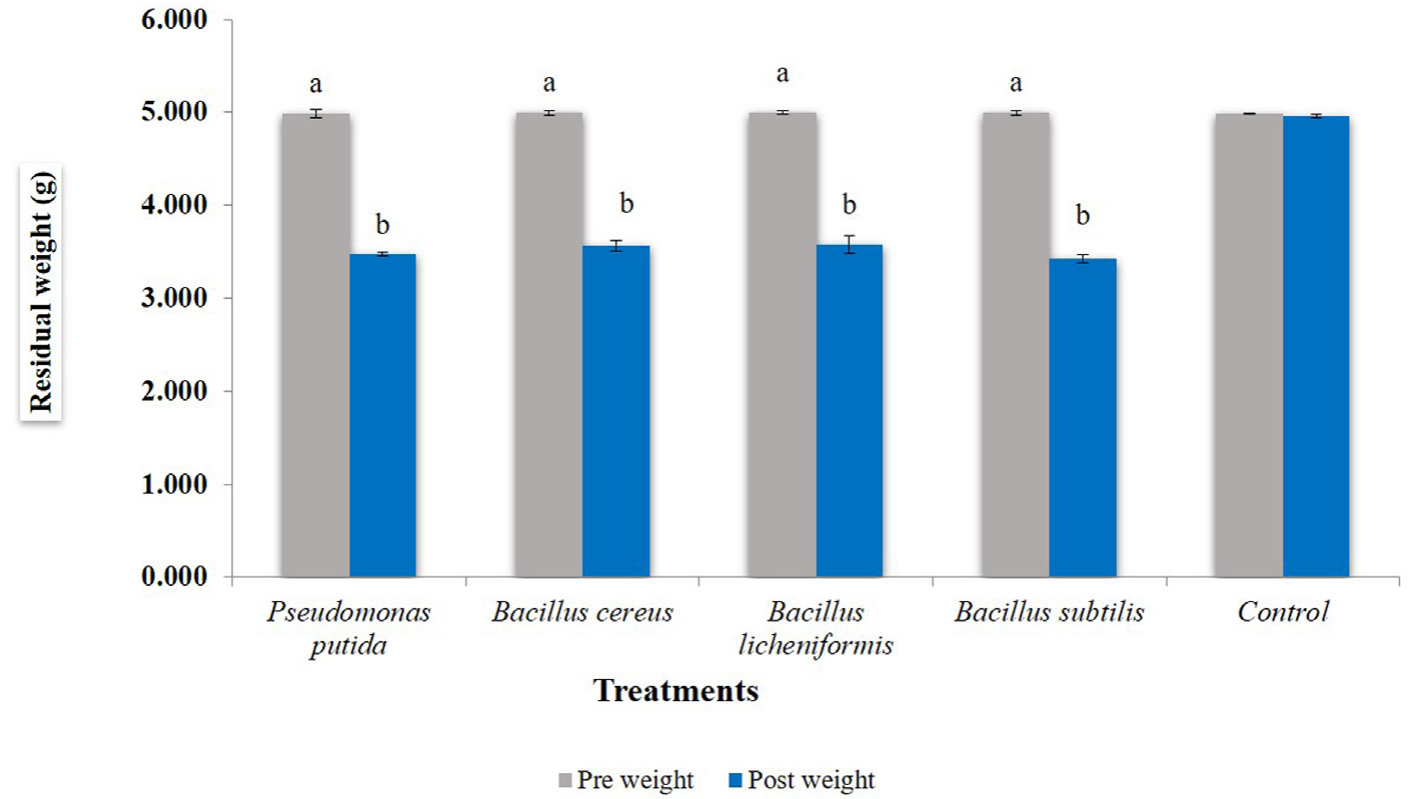
Changes in residual weight (g) of Liquid paraffin within 30 days of incubation time by different bacterial species*.* Bars with similar letters within a treatment are not statistically different (*Tukey* HSD, p < 0.05).

The bacteria were evaluated for residual weight of polyethylene (LDPE) within 45 days of incubation period. Within treatment, a significant reduction in weight of polyethylene was observed at 15 days after treatment (F = 17.167, df = 4, 10, p = 0.000), 30 (F = 15.125, df = 4, 10, p = 0.000) and 45 (F = 31.556, df = 4, 10, p = 0.000) (Table 2). After 45 days of incubation, the highest percent reduction in residual weight was observed in *B. cereus* (38.88%) while the lowest was observed in *P. putida* (26.11%) (Fig. 2). A significant increase in weight reduction of polyethylene was observed with increase in time interval within each bacterial species viz., *B. subtilis* (F = 1.589, df = 2, 6, p = 0.000), *P. putida* (F = 2.632, df = 2, 6, p = 0.000), *B. licheniformis* (F = 4.89, df = 2, 6, p = 0.000) and *B cereus* (F = 8.77, df = 2, 6, p = 0.000) (Fig. 3).

**Table 2:** Effect of different bacterial species grown on nutrient agar on residual weight of low density polyethylene (LDPE) within 45 days of incubation period.

**Treatments**	**Days after treatment**			
	**0**	**15**	**30**	**45**
	**Pre weight (g)**	**Post weight(g)**	**Post weight(g)**	**Post weight(g)**
***Bacillus cereus***	0.0180 ± 0.0000^a^	0.0157 ± 0.0006^a^	0.0140 ± 0.0010^a^	0.0110 ± 0.0010^a^
***Bacillus subtilis***	0.0177 ± 0.0006^a^	0.0167 ± 0.0006^b^	0.0157 ± 0.0006^a^	0.0130 ± 0.0010^a^
***Bacillus licheniformis***	0.0177 ± 0.0006^a^	0.0153 ± 0.0006^a^	0.0140 ± 0.0010^a^	0.0123 ± 0.0006^a^
***Pseudomonas putida***	0.0180 ± 0.0000^a^	0.0170 ± 0.0000^c^	0.0153 ± 0.0006^a^	0.0133 ± 0.0006^b^
***Control***	0.0180 ± 0.0000^a^	0.0180 ± 0.0000^d^	0.0180 ± 0.0000^b^	0.0177 ± 0.0006^c^

Means ± SE bearing similar superscript of small letter in a column do not differ significantly (*Tukey* HSD, p < 0.05). SE = standard error of means.

### 3.3 Surface morphology

The SEM images showed some localized degradation of the polyethylene around the bacterial cells in the bio film (Fig. 1). The treated LDPE film showed rough surface with several cracks and grooves after 45 days of incubation. On the other hand, control samples showed smooth surface under same condition.

### 3.4 Tensile strength of polyethylene

All bacteria showed significant change in tensile strength (t = 5.732, df = 14, p = 0.000) over control. Among all the treatments, *P. putida* (0.633 N/mm^2^) showed the highest tensile strength followed by *B. subtilis* (0.542 N/mm^2^), *B. licheniformis* (0.331 N/mm^2^) and *B. cereus* (0.210 N/mm^2^) over control (0.854 N/mm^2^) (Fig. 2). The treatment with lowest tensile strength has got the highest degradation and vice versa.

### 3.5 Residual weight of liquid paraffin

All bacteria showed significant reduction in weight of liquid paraffin (t = 7.641, df = 14 and p = 0.000) over control (Fig. 3). Among all the treatments, *B. subtilis* was found to be most efficient (3.421 g) followed by *P. putida* (3.471 g), *B. cereus* (3.568 g), *B. licheniformis* (3.576 g) and control (4.90 g) (Fig. 5).

**Fig. 4:**
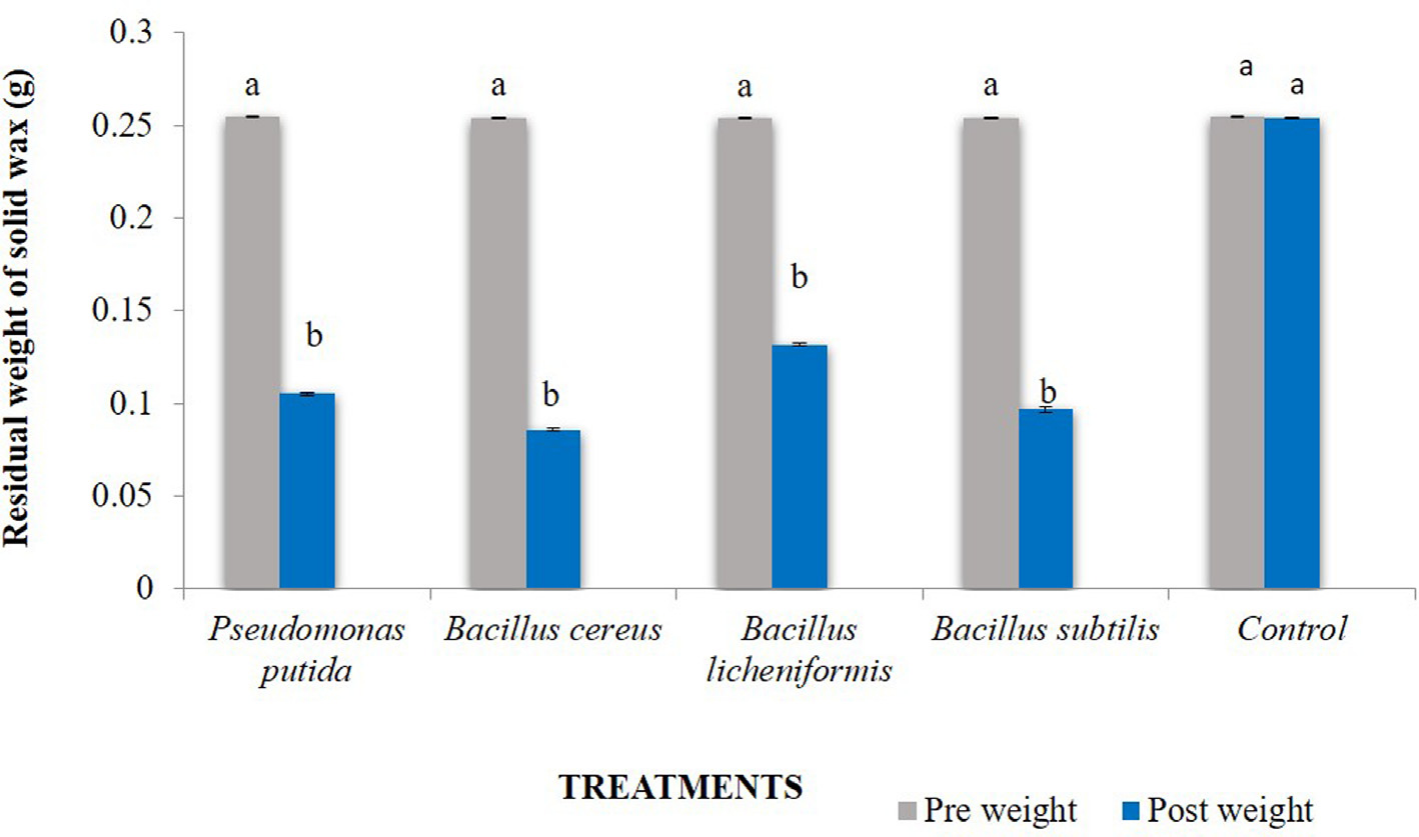
Effect of different bacterial species grown on nutrient agar on residual weight (g) of solid wax after 30 days of incubation period*.* Bars with similar letters within a treatment are not statistically different (*Tukey* HSD, p < 0.05).

**Fig. 5:**
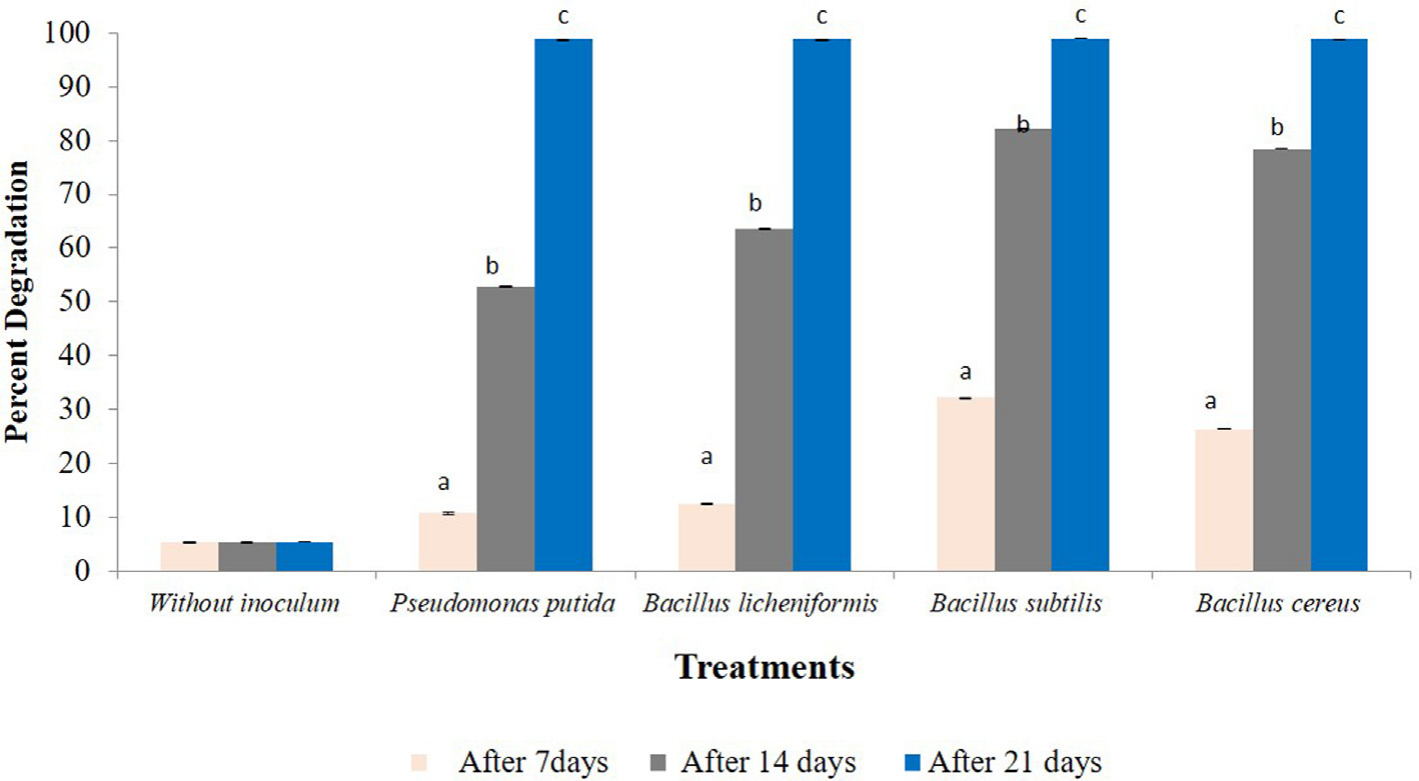
Bioremediation of chlorpyriphos (%) within 21 days of incubation of bacterial inoculum@ (1 × 10^8^ cfu/ml) in soil*.* Bars with similar letters within a treatment are not statistically different (*Tukey* HSD, p < 0.05).

### 3.6 Effect of bacterial species on residual weight of solid wax

The incubated samples containing strains viz., *B. cereus, B. subtilisi, B. licehniformis and P. putida* showed significant reduction in residual weight of solid wax (t = 7.286, df = 14, p = 0.000) as compared to the untreated control. The highest reduction was observed by *B. cereus* (66.53%) followed by *B. subtilis* (62.20%), *P. putida* (58.66%) and *B. licheniformis* (48.42%) (Fig. 4). However, no difference was observed in the residual weight of solid wax in control after 30 days (0%).

### 3.7 Degradation of chlorpyriphos in soil

**Fig. 6:**
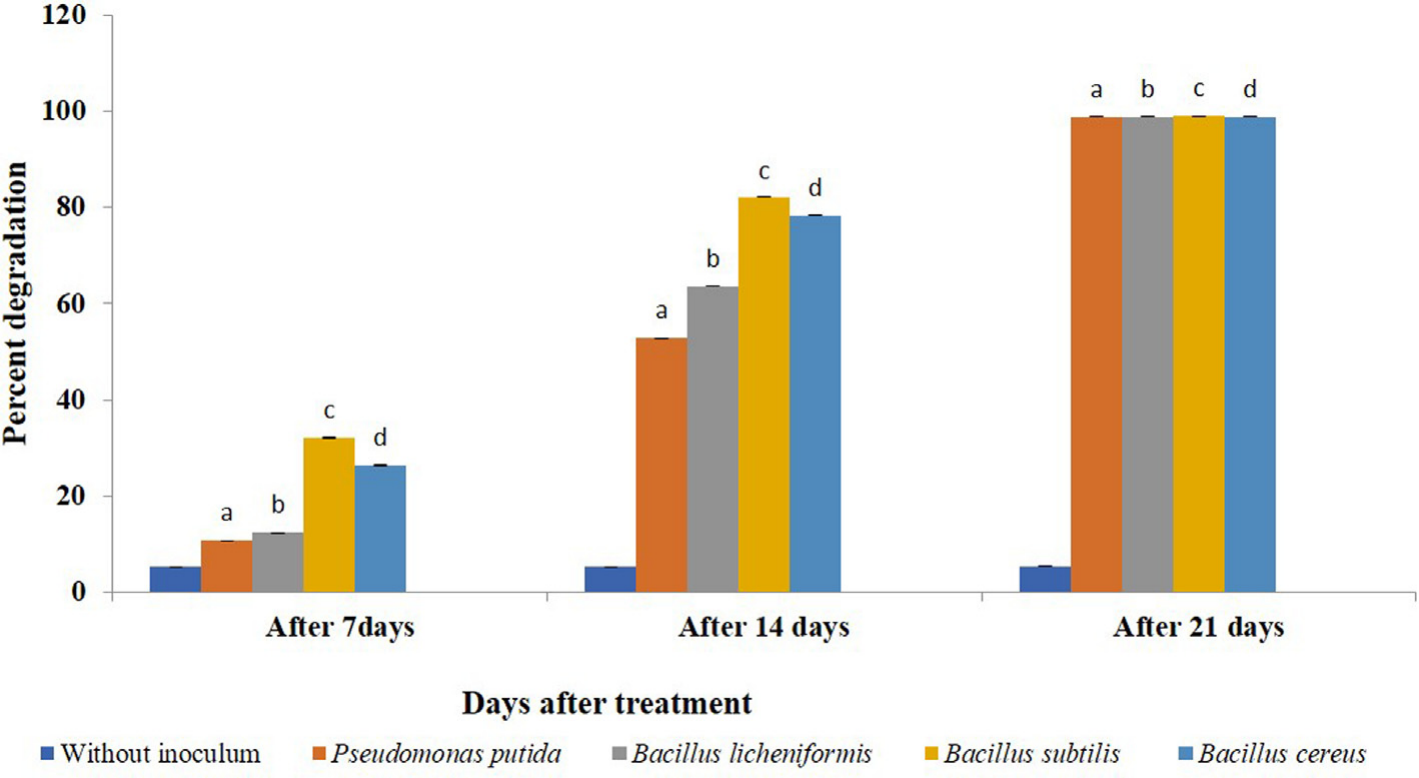
Dissipation rate (%) of chlorpyriphos at different time interval within bacterial species in soil*.* Bars with similar letters within a treatment are not statistically different (*Tukey* HSD, p < 0.05).

All the treatments showed reduction in the amount of chlorpyriphos in the soil. Within a treatment, a significant difference in degradation of chlorpyriphos after 7 days (F = 5.968, df = 4, 10, p = 0.000), 14 days (F = 4.523, df = 4, 10, p = 0.000) and 21 days (F = 1.551, df = 4, 10, p = 0.000) of treatment was observed when compared with control (Table 3, Supplementary 6). While *B. subtilis* proved significantly superior to all other treatments on 7 days and 14 days after treatment. *B. cereus* and *B. licheniformis* were found equally effective at 21 days after treatment*.* The highest degradation was observed in *B. subtilis* (98.98%) after 21 days of treatment (Fig. 5). Across incubation period, the rate of dissipation significantly increased within each bacterial isolate viz., *B. subtilis* (F = 2.399,df = 2,6,p = 0.000), *B. licheniformis* (F = 3.124, df = 2,6, p = 0.000), *B. cereus* (F = 4.196 ,df = 2,6,p = 0.000) and *P. putida* (F = 2.414, df = 2,6,p = 0.000) (Fig. 6).

**Table 3:** Degradation of soil applied chlorpyriphos (µg/ 20 µl) by different bacterial isolates.

**Treatments**	**Chlorpyriphos (µg/ 20 µl)**		
	**Days after treatment**		
	**7**	**14**	**21**
**Without inoculums**	0.606 ± 0 .00000^e^	0.606 ± 0.00000^e^	0.594 ± 0.00000^c^
***Pseudomonas putida***	0.541 ± 0.00153^d^	0.286 ± 0.00058^d^	0.007 ± 0.00000^b^
***Bacillus licheniformis***	0.531 ± 0.00115^c^	0.221 ± 0.00058^c^	0.007 ± 0.00058^b^
***Bacillus subtilis***	0.411 ± 0.00115^a^	0.108 ± 0.00058^a^	0.006 ± 0.00000^a^
***Bacillus cereus***	0.446 ± 0.00321^b^	0.131 ± 0.00058^b^	0.007 ± 0.00058^b^

Means ± SE bearing similar superscript of small letter in a column do not differ significantly (*Tukey* HSD*,* p < 0.05).

### 3.8 Degradation of chlorpyriphos in water as media

**Fig. 7:**
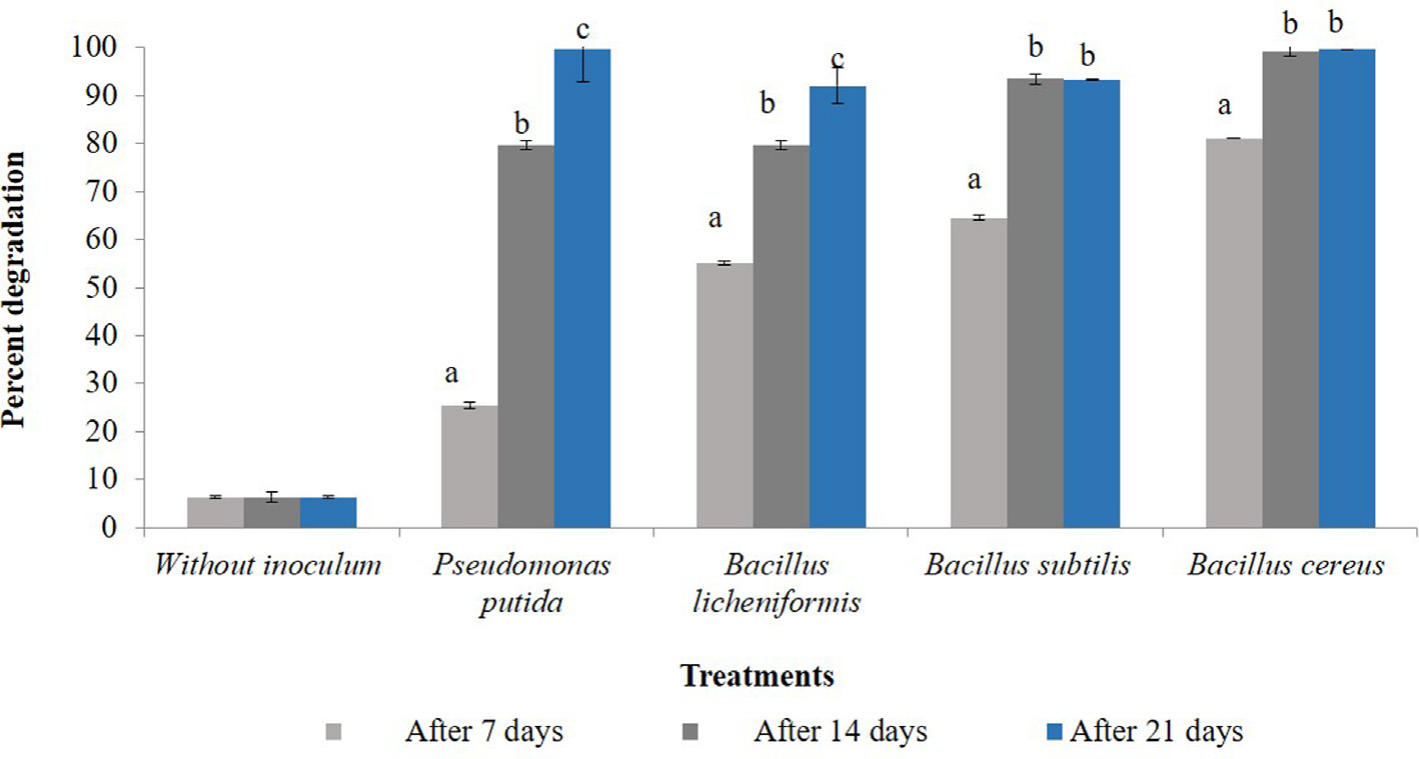
Bioremediation of chlorpyriphos (%) within 21 days of incubation of bacterial inoculum @ (1 × 10^8^ cfu/ml) of water*.* Bars with similar letters within a treatment are not statistically different (*Tukey* HSD, p < 0.05).

**Fig. 8:**
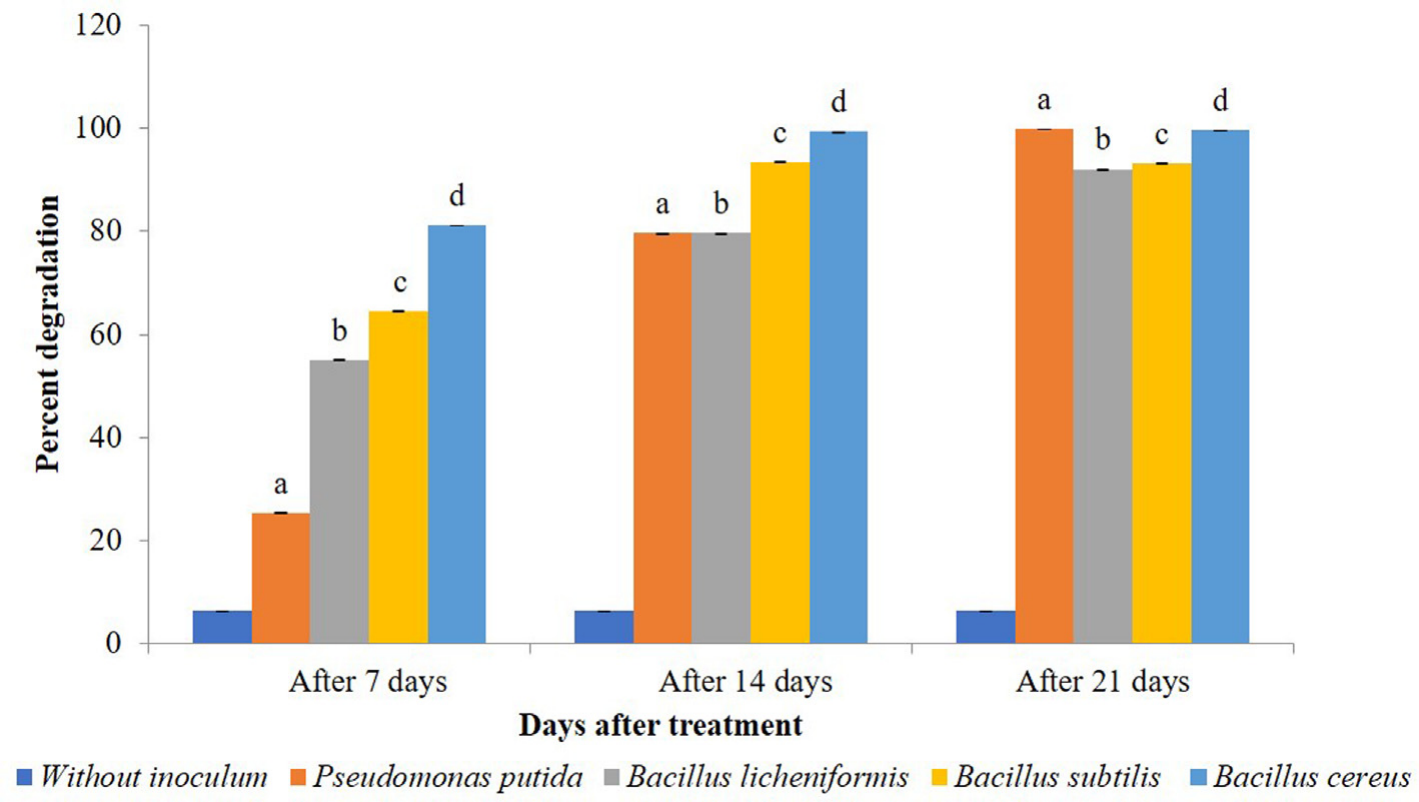
Dissipation rate (%) of chlorpyriphos at different time interval within bacterial species in water*.* Bars with similar letters within a treatment are not statistically different (*Tukey* HSD, p < 0.05).

When compared to control, a significant reduction in the amount of chlorpyriphos was observed after 7 days (F = 3.621, df = 4, 10, p = 0.000), 14 days (F = 416.554, df = 4, 10, p = 0.000) and 21 days (F = 150.994, df = 4, 10, p = 0.000) of treatment. The most efficient bacteria in reducing the amount was *B. cereus* and *P. putida* (99.75%) followed by *B. subtilis* (93.20%) and *B. licheniformis* (92.71%) (Table 4, Fig. 7, Supplementary 7). However, when compared over incubation time the rate of dissipation significantly increased within each bacterial isolate i.e., *B. subtilis* (F = 25.667, df = 2, 6, p = 0.001)*, P. putida* (F = 4.044, df = 2, 6, p = 0.000)*, B. licheniformis* (F = 23.331, df = 2, 6, p = 0.002) *and B. cereus* (F = 2.072, df = 2, 6, p = 0.000) (Fig. 8).

**Table 4:** Degradation of chlorpyriphos in water by different bacterial isolates.

**Treatments**	**Chlorpyriphos (µg/ 20 µl)**		
	**Days after treatment**		
	**7**	**14**	**21**
**Without inoculums**	0.412 ± 0.0000^e^	0.412 ± 0.0000^c^	0.412 ± 0.0000^b^
***Pseudomonas putida***	0.307 ± 0.0064^d^	0.084 ± 0.0036^b^	0.001 ± 0.0012^a^
***Bacillus licheniformis***	0.185^c^ ± 0.0049^c^	0.084 ± 0.00723^b^	0.030 ± 0.0494^a^
***Bacillus subtilis***	0.146 ± 0.00265^b^	0.027 ± 0.0303^a^	0.028 ± 0.0266^a^
***Bacillus cereus***	0.078 ± 0.0010^a^	0.003 ± 0.0025^a^	0.001 ± 0.0010^a^

Means ± SE bearing similar superscript of small letter in a column do not differ significantly (*Tukey HSD* at p < 0.05).

## 4 Discussion

This study aimed to explore the possibility of using endosymbionts for bioremediation of pesticides, wax and polyethylene. Since, mealy bug is well known to escape the pesticidal treatments due to the presence of wax and is also detoxifies the pesticides due to endosymbionts (Sreerag et al., 2014, Kantheti et al., 1996, Munson et al., 1992). Therefore, these endosymbionts could be explored in degradation of insecticides, waxes and polyethylene.

Natural bioremediation has been used for wastewater treatment, but its application in agriculture has immense potential to not only reduce the pesticide in the ecosystem but also the agricultural waste, crop residues and polyfilms. The use of microorganisms to remediate/destroy or to immobilize pollutants from the environment is a recent approach that might be considered an excellent process to remove agrochemicals in water, soil and sediments (Abatenh et al., 2017).

Chlorpyriphos is a popular pesticide that is used extensively for pest control in vegetable and cotton fields. However, chlorpyriphos is highly toxic to mammals and can lead to contamination of soil and water resources. Therefore, it is highly important to identify ways to degrade chlorpyriphos and other pesticides from the environment. The half-life of chlorpyriphos ranges from 60 to 120 days (Howard, 1991). Bioremediation uses the degradation potential of microbes to provide a cost-effective and reliable approach for pesticide abatement. Use of polythene is also increasing day by day. It has been estimated that India's polythene demand is expected to increase by 129% by 2023 (Jaivirk, 2018). The present investigation revealed that endosymbionts use the polythene as a source of carbon by solubilizing them using enzymes. The crop residues are abundant with cellulose, hemicellulose, lignin, pectin and contain a small amount of the diverse group of substances such as protein, waxes and fatty acids. The decomposition of wax rich crop residue like rice straw (2.18%), sunflower residues (3%), Soyabean (0.5%), and sugarcane bagasse (1%) provides major and micronutrients and releases growth promoting substances. Microbial decomposition of crop residues plays an important role in maintaining the soil health by recycling plant nutrients. The biodegradation of these ubiquitous crop residues with suitable microorganism facilitates nutrient from crop residues and maintain sustainable soil fertility and health (Arunkumar et al., 2017). Besides, they were equally effective to degrade the paraffin in nutrient broth media.

Almost all insects have endosymbionts associated with them, required for their normal growth and development (Munson et al., 1992). However, the loss of these microorganisms can result in abnormal development and reduction in the survival of the insect host (Fukatsu and Hosokawa, 2002). Different phytophagous insects are known to be associated with various bacterial species and depending upon their association, these bacteria can be used in biological control of such insects. Similarly, the mealy bug also has some endosymbionts. Isolation of endosymbiotic bacteria has been carried out from the mealy bug (Kantheti et al., 1996, Munson et al., 1992, Gatehouse et al., 2012) and other sap sucking insects (Bing et al., 2013, Ibrahim et al., 2020) and their role for degradation of organophosphate compounds was explored successfully. We have identified *B. subtilis, B. licheniformis, B. subtilis* and *P. putida* from mealybugs (Ibrahim et al., 2020). The results of present findings are in line with the study of several researchers who demonstrated hydrocarbons/Polythene/Paraffin degrading capacity of certain bacterial species like *Bacillus* (Yang et al., 2014), *S. marcescens*, *Pseudomonas aeruginosa* (Wongsa et al., 2004), *Bravibacillus, Achromobacter* and *Pseudomonas*. This may be attributed to the presence of certain biosurfactants, which utilize wax and their compounds as a sole source of carbon (Singer and Finnerty, 1990, Rocha et al., 1992, Arunkumar et al., 2017). For instance, Sakthipriya et al. (2015) demonstrated the production of biosurfactants by *P. aeruginosa* and *P. fluorescens*. Further, biochemical studies also revealed that some microorganisms can also produce degrading enzymes such as lipases to catabolise the long-chain hydrocarbons. They catalyse both hydrolysis and synthesis of esters from glycerol and long chain fatty acids. The presence of extracellular lipase enzyme in a thermophilic bacterium *Bacillus* sp*.* strain L2 was also reported (Shariff et al., 2007). Furthermore, Karthika et al. (2014) reported the presence of hydrocarbon degrading gene in the genome of *P. stutzeri,* which can also accredit to the degradation of hydrocarbons.

The significant dissipation in the amount of chlorpyriphos through the activity of chlorpyriphos degrading bacteria was highly significant by *B. cereus*. Degradation of pesticides by bacteria can be attributed to the presence of various enzymes, including phosphotriesterase, organophosphorus phosphatase (OPP) and organophosphorus hydrolase (OPH). These results are in line with Liu et al. (2012), who demonstrated bacterial degradation of chlorpyriphos by *B. cereus*. It has been reported that *Bacillus* spp., *P. aeruginosa* and *Bravibacillus* sp. are very important in degrading chlorpyriphos, polythene, and paraffin (Wongsa et al., 2004, Yang et al., 2014). Singh et al. (2004) reported that *Enterobacter* sp. has a very strong OPH activity and hydrolyzed 35 mg/l of chlorpyriphos within 24 h when inoculated with 10^6^ cells/ml. The primary mechanism of microbial attack seems to be the hydrolysis of the ester linkages, which destroy the toxicity of the compound by enzymes known as hydrolases, phosphotriesterase, aryl dialkyl phosphatases (Qiao et al., 2003). Though we observed a meagre degradation in control, that could be due to some abiotic factors such as temperature, humidity, etc. (Romeh and Hendawi, 2014). Similarly, a significant increase in the dissipation rate was observed with an increase in time interval. The rate of dissipation of chlorpyriphos increases with incubation time by *Bacillus* spp. (Romeh and Hendawi, 2014). This might be due to the production of metabolites that are consumed further by them. Through the adapted process, the new compounds could induce microorganism to produce the corresponding enzymes or establish a new enzyme system to degrade them.

The degradation of polyethylene by endosymbionts showed a significant reduction in weight of polyethylene sheet after 15, 30 and 45 days of treatment. Our results are in line with earlier reports that showed the biodegradation of Low-Density Polythene (LDPE) by *Pseudomonas* spp. and *Bacillus* spp (Kyaw et al., 2012, Muhonja et al., 2018). This degradation of polyethylene by endosymbionts is attributed to biosurfactants produced by these microorganisms (Vimala and Mathew, 2016). It has been reported that *Streptomyces* isolates SSP 14 showed high bioemulsifer production in culture medium (Soud, 2019). In addition to bioemulsifiers, enzymes such as catalase and lipase produced by microorganisms are involved in the degradation of polyethylene (Frazer, 1994). The microbes attach to the inert surface of polyethylene with the help of enzymes and grow on film by utilizing the LDPE and polymers degraded by mineralization into carbon dioxide, water and methane. The weight reduction of polyethylene by microbes increased with time. Muhonja et al. (2018) observed that weight loss in LDPE sheets increased with time. This might be due to the production of metabolites, which they further consume, through the adapted process, the new compounds could induce microorganism to produce the corresponding enzyme system or establish a new enzyme system to degrade them.

The SEM images showed localized degradation of the polyethylene around the bacterial cells in the biofilm. Also, LDPE film showed a rough surface with cracks. In contrast, the untreated film retained a smooth surface under the same conditions. Further, treated film showed a rough surface with several cracks and grooves after 60 days of incubation. The bacterial biofilm showed a cell like moulded pattern in polyethylene. This might be due to the production of biofilm on the surface of polyethylene, which reduces the hydrophobicity of the polymer, thereby, improving the degradation rate. Such shapes have previously been noticed for biodegradable polymers, e.g., poly-β-hydroxybutyrate (Otake et al., 1995). Skariyachan et al. (2015) observed an alteration in the surface topology of polyethylene films treated with bacterium. Analysis of chemical composition of LDPE films by GC-MS revealed alkanes, aromatic hydrocarbon, chlorocarbon, saturated fatty acids as well as unsaturated fatty acid and other unknown compounds. The degradation of LDPE by microbes was facilitated by the formation of biofilm on the surface, which enables them to break down the high molecular weight polymer into smaller fragments through enzymatic processes (Kyaw et al., 2012). The pieces of LDPE plastic were incubated along with bacterial strains *Serratia* sp. KC1-MRL, *B. licheniformis* KC2-MRL, *Bacillus* sp*.* KC3-MRL and *Stenotrophomonas* sp. for a period of one month. SEM showed discoloration, spots, erosion, and cracking on the surface of polyethylene film (Jamil et al., 2015).

The tensile strength (percentage elongation) was found to significantly reduce with incubation time with a significant reduction after 45 days of incubation. Similar results were reported by (Hanaa et al., 1998) and Lee et al. (1991) showing the tensile strength reduction of polyethylene film after incubation with microorganisms. Jakubowicz et al. (2006) reported that percentage elongation of the LDPE film was reduced after oxidation by monoxygenase enzyme. Nowak et al. (2011) reported that biodegradation reduces the percentage elongation of polyethylene films.

The results indicated that all bacterial species significantly reduced the weight of paraffin wax and the most effective was *B. subtilis*. The degradation of paraffin has been attributed to the biosurfactants and enzymes such as catalase produced by the bacteria (Querioga et al., 2003, Mobaiyen et al., 2013). Environmental liquid paraffin decomposer strains including *Streptomyces lavendulae*, *Bacillus* sp. and *S. paracox* that decompose 70% of paraffin were identified by Mobaiyen et al. (2013).*P. putida* is among the most extensively studied alkane-degrading bacteria, which catalyses the hydroxylation of linear and branched aliphatic, alicyclic, and alkyl aromatic compounds. This strain is mostly known for its efficient utilization of medium and lengthy carbon chain alkanes from C12 to C22 (van Beilen et al., 2001). Furthermore, the bacteria decrease the density and viscosity of liquid paraffin. *B. subtilis* decreased density and viscosity of paraffin wax more than *B. licheniformis*, which represents the conversion of long chain alkanes to short-chain alkanes (Sadeghazad and Ghaemi, 2003).

A significant reduction in the amount of wax was by *B. cereus* compared to the rest of the strains. Various wax degrading eubacteria genera, *Pseudomonas*, *Alcaligenes*, *Micrococcus*, *Nocardia, Corynebacteria*, Arthrobacter, *Bacillus*, *Rhodococcus* and *Proteus* have been reported to decompose waxy rich residues (Arunkumar et al., 2017). This has been attributed to the production of monoxygenase enzymes and biosurfactants by bacterial isolates (Singer and Finnerty, 1990, Rocha et al., 1992, Mahendra and Alvarez-cohen, 2006, Arunkumar et al., 2017). Sakthipriya et al. (2015) demonstrated that biosurfactant producing *P. aeruginosa* and *P. fluorescens* use waxes as a sole source of carbon. Microorganism can produce degrading enzymes such as lipases to catabolise the long-chain hydrocarbons such as waxes. Fairolniza et al. (2007) reported that a thermophilic bacterium *Bacillus sp*. strain L2 produces an extracellular lipase enzyme to use long chain alkanes (wax).

## 5 Degradation of chlorpyriphos in soil

A significant reduction in the total amount of chlorpyriphos in soil was observed at an interval of 7, 14 and 21 days after treatment by the bacterial isolates. The degradation of chlorpyriphos is attributed to extracellular enzymes (phosphatases, amidases and laccases) secreted into the soil, which can act on the molecule as a substrate. Biodegradation of chlorpyriphos and diazinon organophosphates up to 88% by *Acinetobacter* and *Pseudomonas* sp. isolated from contaminated agricultural soils has been reported by Amani et al. (2019). The microbial hydrolysis of organophosphorus pesticides by *P. putida* and *Flavobacterium* sp. is carried by membrane bound enzymes. The most widely studied bacterial enzyme is the OPH, which hydrolyses the organophosphorus insecticide. (Allard and Neilson, 1997). After 21 days of treatment, all bacterial isolates showed the same level of degradation, which may be because soil acts as a good media for growth and development of all these bacteria. A minute quantity of chlorpyriphos was degraded in the control, which was without inoculum, which could be due to some abiotic factors, such as pH, moisture, temperature, etc. (Cink and Coats, 1993, Racke, 1993). Kumar (2011) observed that using soil slurry as medium, bacterial isolate RCC-2 was found to be most efficient with 21, 37, 54 and 77% of chlorpyriphos degradation in 5, 10, 15 and 30 days of treatment duration, respectively.

A significant reduction was observed in the amount of chlorpyriphos due to the treatment with bacterial isolated and among all bacteria, *B. cereus* and *P. putida* were found to be most effective. *B. cereus* has been reported to have high chlorpyriphos degradation capability (Liu et al., 2012). *Pseudomonas* is a diversified genus possessing a series of catabolic pathways and enzymes involved in pesticide degradation. *P. putida* has been reported to be more efficient in chlorpyrifos degradation by a rate of 90% (Gilani et al., 2016).

The degradation of chlorpyriphos might be due to OPP, and OPH enzymes present in both the intracellular and the extracellular fractions of different bacterial species (Qiao et al., 2003). The rate of degradation increases with time interval, which may be due to the growth of bacterial isolates and production of some metabolites, which they further consume.

## Declaration of Competing Interest

The authors declare that they have no known competing interests financially or personally with other people or organizations that could inappropriately influence this work.
