# Supplementary material for: Degradation of chlorpyriphos and polyethylene by endosymbiotic bacteria from citrus mealybug
Source: Saudi J Biol Sci. 2021 Mar 27;28(6):3214–24. doi: 10.1016/j.sjbs.2021.03.058 (PMC8176133; doi:10.1016/j.sjbs.2021.03.058)
Supplement: Supplementary data 1 [file mmc1.docx]

**A**

**C**

**B**

**A**

**C**

**B**

**Supplementary 1.** Chromatogram on degradation of chlorpyriphos by *Bacillus cereus* after incubation on, A: 7^th^ days after incubation, B: 14^th^ days after incubation, C: 21^st^ days of incubation

**A**

**C**

**B**

**Supplementary 2.** Chromatogram on degradation of chlorpyriphos by *Bacillus licheniformis* after incubation on, A: 7^th^ days after incubation, B: 14^th^ days after incubation, C: 21^st^ days of incubation

**A**

**C**

**B**

**Supplementary 3.** Chromatogram on degradation of chlorpyriphos by *Bacillus subtilis* after incubation on, A: 7^th^ days after incubation, B: 14^th^ days after incubation, C: 21^st^ days of incubation

**A**

**C**

**B**

**Supplementary 4.** Chromatogram on degradation of chlorpyriphos by control after incubation on, A: 7^th^ days after incubation, B: 14^th^ days after incubation, C: 21^st^ days of incubation

k

**A**

**C**

**B**

**Supplementary 5.** Chromatogram on degradation of chlorpyriphos by *Pseudomonas putida* after incubation on, A: 7^th^ days after incubation, B: 14^th^ days after incubation, C: 21^st^ days of incubation

A

B

E

C

D

**Supplementary 6.** Chromatogram on degradation of chlorpyriphos in soil by different bacteria and control A: *Bacillus cereus* B: *Bacillus licheniformi*s C: *Bacillus subtilis* D: *Pseudomonas putida* E: Control

A

B

E

C

D

**Supplementary 7.** Chromatogram of degradation of chlorpyriphos in water by different bacteria and control A: *Bacillus cereus* B: *Bacillus licheniformi*s C: *Bacillus subtilis* D: *Pseudomonas putida* E: Control
